# “Gold” Pressed Essential Oil: An Essay on the Volatile Fragment from* Citrus* Juice Industry By-Products Chemistry and Bioactivity

**DOI:** 10.1155/2017/2761461

**Published:** 2017-10-04

**Authors:** V. N. Kapsaski-Kanelli, E. Evergetis, A. Michaelakis, D. P. Papachristos, E. D. Myrtsi, S. D. Koulocheri, S. A. Haroutounian

**Affiliations:** ^1^Department of Nutritional Physiology and Feeding, Agricultural University of Athens, Iera Odos 75, 11855 Athens, Greece; ^2^Benaki Phytopathological Institute, 8 S. Delta Str., 14561 Athens, Greece

## Abstract

Present essay explores the potentials of* Citrus* juice industry's by-products as alternative bioactive natural products resources. Four crude Cold Pressed Essential Oils (CPEOs), derived from orange, lemon, grapefruit, and mandarin, were studied. All CPEOs were subjected to water distillation, in order to obtain the volatile fragment, which was further fractionated with respect to distillation period in two parts, concluding to eight samples. These samples along with the four original CPEOs were assessed in relation to their phytochemical content and their repellent and larvicidal properties against Asian Tiger Mosquito. The volatiles recovery rates ranged from 74% to 88% of the CPEO. Limonene presented a significant increase in all samples ranging from 8% to 52% of the respective CPEO's content and peaked in mandarin's 2nd volatile fragment which comprised 97% of the essential oil. The refinement process presented clear impacts on both bioassays: a significant increase in larvicidal potency was observed, annotated best by the improvement by 1100% and 1300% of the grapefruit volatile fractions; repellence testing provided only one significant result, the decrease of landings by 50% as a response to mandarin's second volatile fraction. The applied methodology thus may be considered for the improvement of* Citrus* juice industry's by-products chemistry and bioactivity.

## 1. Introduction

Among agricultural commodities and the consequent industries,* Citrus* fruits hold a significant position. According to Food and Agricultural Organization [1], global* Citrus* fruit production in the year 2014 reached approximately 140 Mt, 60% of which were oranges. Worldwide, it is estimated that annually over 30% of* Citrus* fruits produced (40% of oranges) are being processed by the food industry to produce mainly juice based products. This endeavor is generating a considerably high amount of by-products that can be potentially used as a biorefinery raw material [2].

The residues of citrus juice production consisted of peel, pulp seeds, and whole citrus fruits that do not meet the quality requirements, while only 50% of the fresh fruit's mass is transformed into juice [3]. In general, these citrus juicing by-products have an insignificant economic value, even though they are rich in soluble sugars, cellulose, hemicellulose, pectin, flavonoids, and essential oils [2, 4]. Among them the Cold Pressed Essential Oil (CPEO) is widely used by the food, beverage, cosmetic, and pharmaceutical industries as flavoring and fragrance agent due to its characteristic aroma profile [5]. This utilization as industrial raw materials has created a market for these CPEOs in which their price escalation is 6 to 7€ per kg for grapefruit; 8 to 12€ per kg for orange; 12 to 15€ per kg for mandarin; 30 to 35€ per kg for lemon [6]. CPEOs consisted of volatile and nonvolatile fractions that are composed of more than 200 compounds [7]. The volatile fraction, which represents an 85% to 99% of the CPEO, is well characterized in the literature [8]. Phytochemicals commonly found in* Citrus* CPEOS are monoterpene and sesquiterpene hydrocarbons, their oxygenated derivatives and aliphatic aldehydes, alcohols, and esters. The main volatile compound of* Citrus* essential oils is D-limonene, a nonoxygenated monoterpene derived by the combination of two isoprene units [6]. The CPEOs obtained as by-products from the* Citrus* juice industry are recovered in yields ranging from 0,4 to 0,6 mL per kg [6]. However, CPEOs are characterized by high percentages of nonvolatile residues, which contain hydrocarbons, sterols, fatty acids, waxes, carotenoids, coumarins, psoralens, and flavonoids [8].

D-Limonene is one of the world's most widespread terpenes constituting up to 90–95% of the orange peel oil and 75% of lemon peel oil. The worldwide annual production of D-limonene is over 70 million kg and rising fast [9]. In addition to its flavor and fragrance properties, this phytochemical is used in a broad variety of consumer products due to its physicochemical properties. For example, D-limonene is being used as a nontoxic solvent in oleochemical, wax, resin, paint, and glue industrial preparations or as a valuable renewable biosolvent, an alternative to hazardous petroleum solvents [10]. Another major application of D-limonene is its application as a cleaning agent replacing various environmentally unfriendly cleaning agents such as toluene, hexane, and chlorinated organic solvents [11]. Due to its low price, D-limonene is an attractive starting compound for the biotechnological production of industrially relevant fine chemicals and flavor compounds with identical carbon skeletons, such as carveol, carvone, and perillyl alcohol [12].

Herein we present the outcome of our study concerning the sustainable valorization of the* Citrus* juice industry CPEO by-products incorporating a binary approach. This biorefinery method focuses on the retrieval and simultaneous fragmentation of the CPEOs volatile fraction aiming at the increase of the (a) content of D-limonene, a fine chemical with distinct exploitation potentials and (b) bioactivity of the processed CPEO volatile fragments. As a target organism for the bioactivity assessment, the Asian Tiger Mosquito was chosen,* Aedes (Stegomyia) albopictus* (Skuse 1894) (Diptera: Culicidae), which is currently considered as the most invasive mosquito species in the world [13] with great public health importance since it is a confirmed vector of Yellow Fever [14], dengue fever [15], Chikungunya fever [16], and Zika [17] viruses.

## 2. Materials and Methods

### 2.1. Materials

The original material of the study consisted of the CPEOs derived from the industrial processing [18] of the following four different* Citrus* species, (a) Orange,* Citrus sinensis* (L.) Osbeck; (b) Lemon,* C. limon* (L.) Osbeck; (c) Grapefruit,* C. paradisi* Macfad.; (d) Mandarin,* C. reticulata* Blanco, which were kindly provided by the industry of fruit juices Christodoulou Bros SA. All sampling details are included in Table 1.

### 2.2. Methods

#### 2.2.1. Isolation and Fragmentation of the CPEO Volatile Fraction

The four-industrial CPEOs were subjected to conventional hydrodistillation. All distillations were performed with 3 L of H_2_O in a modified Clevenger apparatus for 3 hours. Four EOs were obtained as the initial fragments (first 20 min) of the hydrodistillation of these industrial by-products and another four were isolated from the remaining fragments (consequent 2 h and 40 min). The essential oil yields are included in Table 1.

#### 2.2.2. Gas Chromatography-Mass Spectrometry (GC/MS)

The GC/MS analyses were performed on a Agilent Technologies 7890A Gas Chromatograph, equipped with a HP 5MS 30 m × 0.25 mm × 0.25 *μ*m film thickness capillary column, connected with an Agilent 5957C, VL MS Detector with Triple-Axis Detector system operating in EI mode, and He as the carrier gas (1 mL/min). The initial column temperature was 60°C and heated gradually to 280°C with a 3°C/min rate. The identification of the compounds was based on comparison of their Retention Indices (RI) obtained, using various n-alkanes (C_9_–C_24_) and their EI-mass spectra were compared with the NIST/NBS, Wiley libraries spectra, and literature [19, 20]. Additionally, the identity of the indicated phytochemicals was confirmed by comparison with available authentic samples. All authentic samples utilized for the identification of EOs compounds were obtained from Sigma-Aldrich, except for germacrene D and *α*-thujene, which had been isolated in the context of previous studies.

#### 2.2.3. Rearing* Ae. albopictus* in the Laboratory

Larvae and adults of* Ae. albopictus* were obtained from a laboratory colony which was maintained at 25 ± 2°C, 80% relative humidity and photoperiod of 16 : 8-h light/dark (L/D), in the laboratory of the Benaki Phytopathological Institute, Kifissia, Greece. The adults were kept in wooden frame cages (33 × 33 × 33 cm) with a 32 × 32 mesh, with easy access to 10% sucrose solution on a cotton stick. Females were fed with fresh chicken blood with Hemotek^©^ blood feeding system. The larvae were reared in tap water-filled cylindrical enamel pans with diameter of 35 and 10 cm deep covered by fine muslin. Approximately 400 larvae were fed in excess with powdered fish food (JBL Novo Tom 10% Artemia) in each pan until the emergence of adults. Adult mosquitoes were often collected with a mouth aspirator and transferred to the rearing cage. Plastic beakers with 100 ml water and strips of moistened filter paper were provided in the cage for oviposition. The eggs were kept damp for a few days and then placed in the pans for hatching [21].

#### 2.2.4. Larvicidal Bioassays

The larval mortality bioassays were carried out according to the test method of larval susceptibility as recommended by the World Health Organization [22] with modifications. Sufficient amounts of each compound were transferred to a vial and the residual solvent was removed under high vacuum. Stock solutions of 10% (w/v) in dimethyl sulfoxide (DMSO) were prepared for each testing material. Twenty late third-to early fourth-instar mosquito larvae were placed in 2% (v/v) aqueous solution of DMSO (98 ml of tap water plus 2 ml of DMSO), followed by the addition of 29 *μ*l of the tested material solution. The aqueous solutions were then gently shaken for homogenization [21]. Five replicates per dose were tested and a treatment with 98 ml of tap water and 2 ml of DMSO was included in each bioassay as the control. The mortality rates of the essential oils tested were arbitrary classified to “low,” “moderate,” and “very good” if the mortality rates ranged between 0–50%, 50–80%, and 80–100%, respectively.

#### 2.2.5. Repellent Activity Bioassay

For the repellent activity of the essential oils, the assessment was based on the human landing counts [23, 24]. The study was conducted into a cage (33 × 33 × 33 cm) with a 32 × 32 mesh and with a 20 cm diameter circular opening fitted with cloth sleeve. Each cage contained 100 adult mosquitoes (sex ratio, 1 : 1), 5 to 10 days old, starved for 12 h at 25 ± 2°C, and 70–80% relative humidity. A plastic glove with an opening measuring of 5 × 5 cm was employed for all the bioassays. Different doses (from 0.05 to 1 *μ*lcm^−2^) for DEET were applied and it was found that the lowest dose, where zero landings were counted, was ≈0.2 *μ*lcm^−2^. All testing materials were applied on paper (Whatman chromatography paper) of 24 cm^2^ total area and tested at two doses: 50 *μ*l (“low,” ≈0.2 *μ*lcm^−2^ of testing material) and 100 *μ*l (“high,” ≈0.4 *μ*lcm^−2^ of testing material) of 100 *μ*g*μ*l^−1^ stock solution. Control treatments without the components and with DEET were also included for the repellency tests as standards (control and positive control, resp.). Each treatment was repeated eight times and four human volunteers were used [21, 25].

#### 2.2.6. Data Analysis

Larvicidal effect was recorded 24 h after treatment. Data obtained from each dose-larvicidal bioassay (total mortality, mgl^−1^ concentration in water) were subjected to probit analysis in which probit-transformed mortality was regressed against log_10_-transformed dose; LC_50_, LC_90_ values and slopes were calculated (SPSS 11.0).

Data concerning the repellency of the samples (mosquito landings) were analyzed using Kruskal-Wallis test. When significant differences were detected, Mann-Whitney *U* tests were carried out for pairwise comparison. Bonferroni correction was applied to correct for 66 pairwise comparisons leading to an adjusted *a* = 0.0006 [25].

## 3. Results

The volatile fractions of the four* Citrus taxa* presented essential oil yields and recovery rates as percent of the relative industrial CPEO, which are included in Table 1. From the four CPEOs studied orange exhibited the highest volatile recovery rate (0,880), whereas lemon exhibited the lowest (0,740). In all cases, the essential oil yields were higher during the first 15 min of the hydrodistillation (fragment 1) of the CPEOs.

### 3.1. Phytochemical Assessment

The detailed qualitative and quantitative analytical data of the main volatile constituents of the CPEOs have been summarized in Table 2. It must be noted that 30 different phytochemicals, representing 73.89% to 99.98% of the respective samples were identified by combined GC and GC/MS analyses as constituents of the samples studied. The investigated samples were found to contain mainly monoterpenes, mostly cyclic, and only occasionally aliphatic. More specifically, particularly, in the case of orange, grapefruit, and mandarin, D-limonene was by far the most abundant component (up to 97.79%), while compounds like myrcene and *α*-pinene where found in lower percentages. On the contrary, in the case of lemon EOs, D-limonene was found in lower percentages (up to 56.50%) followed by *β*-pinene, *γ*-terpinene, myrcene, *α*-pinene, neral, and citral, among others.

Similar findings of a previous study on the CPEOs derived from industrial cold-pressing of fresh fruit peels of orange, mandarin, and lemon are in accordance with the above-mentioned results, where D-limonene was the most abundant compound in all fruits with concentrations reaching 85.5% in orange, 74.38% in mandarine, and 59.1% in lemon [26]. Ahmad et al. [27] investigated the chemical composition of citrus essential oils collected by cold-pressing of shredded fruit peels. According to their findings, the abundance of D-limonene in two sweet orange varieties was 61.08% and 76.28%, in grapefruit 86.27%, and in lemon 53.61%. According to previous results on the chemical composition of lemon CPEO, D-limonene exhibited the highest percent (75.68%) followed by *β*-pinene (8.7%) and *γ*-terpinene (7.19%) [28]. Concerning mandarine, Sawamura et al. [29] studied the CPEO retrieved by a hand-pressing method of whole fruits and peels and resulted in a high percent of D-limonene (80.3%). Thus, few studies investigate the chemistry of citrus CPEOs, while no previous study exists concerning the CPEOs produced as by-products by the citrus juice producing industry. It is important to notice that the quantitative results of the previous studies mentioned above refer to uncorrected percentages of chemical constituents due to the presence of nonvolatile compounds in the CPEOs that are not detected by the analytical instruments used.

**Figure 1:**
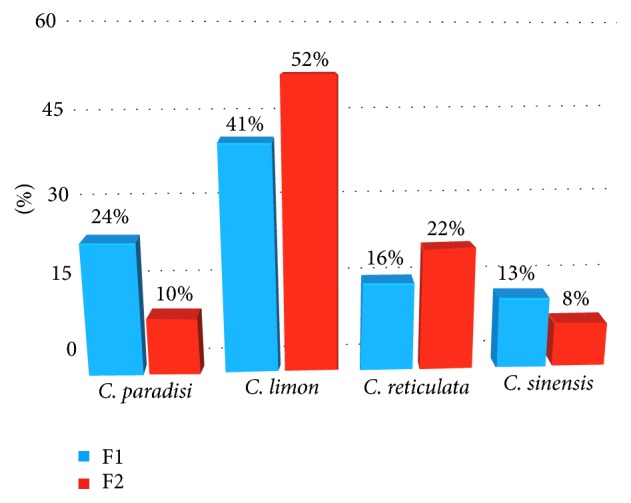
D-Limonene increase in the refined samples, expressed as percentage of the relevant CPEO limonene content.

The applied herein process consisted of the simultaneous tasks of isolation and fractionation of the CPEOs volatile content, which was evaluated for first time presenting intriguing results in respect to the produced essential oils major compounds (above 3% of the essential oil). More specifically, the D-limonene concentration was increased in all cases in both hydrodistillation fragments (1 and 2) in comparison with its original occurrence in the respective CPEOs as indicated in Figure 1. In grapefruit, the percent of D-limonene in the CPEO (72.35%) reached 89.63% in fragment 1 and 79.56% in fragment 2 of the hydrodistillation, whereas other main compounds with increased abundancy in fragment 2 were *α*-terpineol (3,19%) and *β*-caryophyllene (4,26%). For lemon, the abundance of D-limonene was increased from 37.22% in the CPEO to 52.66% in fragment 1 and 56.50% in fragment 2, while a noticeable increase of *β*-pinene (19.16%), *α*-pinene (5.08%), and *γ*-terpinene (13.86%) content is also observed in fragment 1. A sharp increase of D-limonene content is observed after the hydrodistillation of the mandarin CPEO. Specifically, the presence of D-limonene reached 92.75% and 97.79% in fragments 1 and 2 of the hydrodistillation, respectively, as compared to its initial abundance in the CPEO which was determined as 80.06%. Other constituents with increased abundance in the first fragment of the hydrodistillation are myrcene (4.28%),* a*-pinene (1.74%), and sabinene. Finally, for oranges, the D-limonene content was increased in the first fragment of the hydrodistillation, reaching the 95.01%. In the second fragment, an increase of the content of D-limonene, myrcene, and* a*-terpinolene was observed.

### 3.2. Bioactivity Assessment

#### 3.2.1. Larvicidal Bioassays

*Citrus* based essential oils (EOs) have already been pinned as potent population control agents against* Aedes* sp., by Khan and Akram [30], who suggested the relevant consideration of EOs from* C. grandis, C. sinensis, C. paradisi, C. reticulata, C. limon*, and* C. aurantium* but also identified the necessity for the design and implementation of relevant field trials. Four years earlier, in 2009 Michaelakis et al. [31] reported the increased larvicidal activity of the EOs derived from the dried peels of* C. sinensis, C. limon,* and* C. aurantium* against* Culex pipiens* (Diptera: Culicidae). Since then the toxicity of* Citrus *EOs has been confirmed against two* Culex *sp.* taxa, Cx. pipiens* [31] and* Cx. quinquefasciatus* [32]; two* Anopheles *sp.* taxa, An. labranchiae *[33] and* An. stephensi* [34]; two* Aedes *sp.* taxa, Ae. albopictus *[21, 35] and* Ae. aegypti* [36, 37]. Adaptation of these results as a starting point for scientific discussion was implemented after the performance of an insect toxicity sketch, which included as complementary targets the* larvae* of* Tribolium castaneum* Herbst (Coleoptera: Tenebrionidae) investigated by Bilal et al. [38] and* Spodoptera frugiperda* (Lepidoptera: Noctuidae) performed by Villafañe et al. [39].

Since the larvicidal properties of* Citrus* EOs are solidly established, the serious question phrased by Khan and Akram [30] concerning the necessity for field trials implementation remains. The latter require the availability of a large volume of a consistent quality EO. An intriguing answer to this question was suggested in 2011 by Did et al. [35] that employed the EOs isolated from the* Citrus* juice industry solid wastes, which though potent as a source required additional infrastructure development and additional production stages to become broadly available. Our approach herein refers to the exploitation of readily available by-products of the* Citrus* juice industry that are defined as the CPEOs, all four of which are assessed on their toxicity per se, and after refinement, for first time herein.

**Figure 2:**
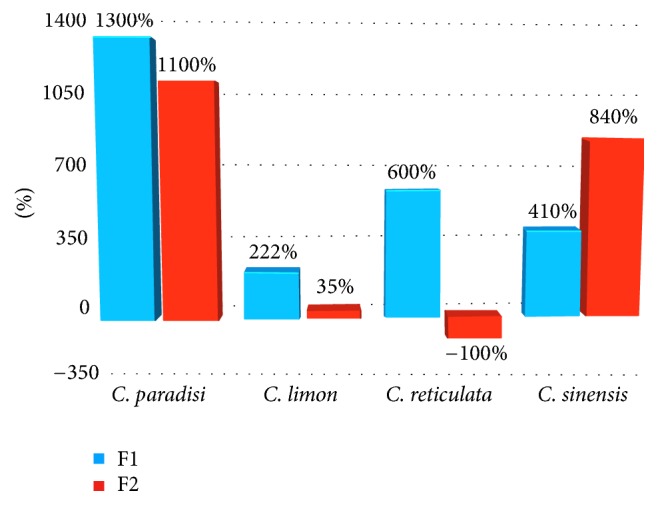
Larvicidal toxicity of the refined samples, expressed as percentage of the relevant CPEO toxicity.

The larvicidal bioassay results are cumulated in Table 3, where the mean percentage (±s.e.) of dead larvae is presenting and indicates results 24 h after the implementation of the testing material. The last consisted of the 4 CPEO and 8 EO samples of Table 1. From the four CPEOs tested those from grapefruit and mandarin were found to be of insignificant toxicity, the one from orange exhibited a mild toxicity, and this from lemon presented a moderate toxicity, justifying thus the depreciated value of the respective by-products. The applied process though proved significant by improving the larvicidal properties in seven of the eight in total, processed samples, as indicated in Figure 2. Even the percentages of Figure 2 indicate an impressive increase of toxicity for the two grapefruit EOs, as most potent proved the lemon first volatile fraction (C05) and the second volatile fraction from orange (C12), exhibiting, respectively, 74% and 94% larvae mortality. Among the eight volatile fractions, only the second volatile fraction of mandarin presented no toxicity (C9). This sample presented a unique phytochemical profile consisting of the highest percentage of D-limonene (97,8%).

These preliminary results from the 12 samples tested did not provide any relation between the phytochemical content or the fluctuation of the major compounds and the larvicidal pattern.

The combined study of larvicidal results in conjunction with the low toxicity of mandarin second volatile fraction is indicative that the accompanying the limonene phytochemicals could be attributed to the increase of toxicity. Previous studies [21] indicated that some commonly found substances in EOs with significant larvicidal activity are* a*-pinene, 3-carene, (*R*)-(+)-limonene, myrcene, and terpinen-4-ol. The increased toxicity that is recorded in all samples containing amounts of myrcene exceeding the limit of 3% may partly explain this toxicity pattern. In either case, the obtained results could justify the application of the simultaneous volatile fraction isolation and fragmentation.

#### 3.2.2. Repellence Bioassays

The repellent properties of* Citrus* derived EOs have been studied and confirmed in a band wider than the larvicidal band of target organisms. In these, the following is included: six Coleoptera,* Periplaneta americana* [40, 41],* P*.* fuliginosa* [41],* Blattella germanica* [40, 41],* Neostylopyga rhombifolia* [40],* Tribolium castaneum* [38, 42], and* Sitophilus zeamais* [43]; the aphid* Tetranychus urticae *[44]; seven Diptera,* Loutzomyia youngi* [45],* Simulium damnosum* [46],* Aedes albopictus* [21, 47],* Ae. aegypti* [48],* Anopheles dirus* (ibid.),* An. stephensi *[34], and* Culex quinquefasciatus* [49]. Within this rough background and based upon these significant research efforts, the present study focused on the investigation of the four-industrial origin CPEO and the eight derived volatile fragments, which is a novelty of the study.

The results summarized in Table 4 present the mean number of landings for five minutes. All four CPEOs presented a low to moderate repellent activity. The applied process of CPEOs refinement did not provide any significant results on the respective repellence activity. Specifically, the orange volatile fractions (C11, C12) exhibited almost the same figures with the respective CPEO (C10) landing averaging to around 50; the application of grape fruit (C02, C03) and lemon (C05, C06) volatile fractions presented an increase of landings compared with the relevant CPEOs (C01, C04), therefore presenting a significant decrease in correlation with their repellent properties; on the other hand, mandarin volatile fragments presented a differentiated performance since both the first and second fragments improved the CPEO's (C07) repellence exhibiting less landings, by 50% for the second fragment (C09) and by 22% for the first fragment (C08). As potential factor for the observed differentiation, the phytochemicals contained in the colorant fragment of the CPEOs may be considered, the lack of which is the major difference between the CPEO and the relative EOs. Those results agree with previous studies indicating that most hydrocarbons (e.g.,* a*-pinene) had a lower repellent efficacy against adult mosquitoes compared to aldehydes, oxides, or alcohols [50].

## 4. Conclusions

In conclusion, the current work exploiting invaluable industrial by-products demonstrated a protocol that can provide D-limonene of analytical grade (97%), revealing thus in the form of the mandarin second volatile fragment an alternative source for the retrieval of this valuable molecule.

Industrial by-products as natural insecticides could be a promising tool especially for targeting mosquito larvae. The lack of any significant difference in the phytochemical content of the mandarin three samples, with the simultaneous differentiation of bioactivity in both larvicidal and repellency bioassays, indicates that the root cause may not be detected in the volatile fragment components and there are some factors that need more investigation to extract useful conclusions such as enantioselectivity of major and minor ingredients [21, 51, 52].

## Figures and Tables

**Table 1:** The four *Citrus* fruits samples *taxon* attribution, definition, and recovery yield (CPEO = Cold Pressed Essential Oil; F1 = fragment 1 (first 15 min); F2 = fragment 2 (consequent 2 h and 45 min). ^*∗*^Volume of the respective CPEO. ^*∗∗*^In the case of CPEO, the industrial figure corresponds to % W/W of produced juice [6]).

*Taxon*	Code	Sample definition	Volume distilled^*∗*^, recovered (mL)	Yield (% V/V)^*∗∗*^
*C. paradisi*	C 01	CPEO	50,0	0,4–0,6%
	C 02	F1	23,2	46,4%
	C 03	F2	18,5	37,0%
	Total vol. fr.		41,7	83,4%

*C. limon*	C 04	CPEO	20,0	0,4–0,6%
	C 05	CPEO vol. fr. 1	8,3	41,5%
	C 06	CPEO vol. fr. 2	6,5	32,5%
	Total vol. fr.		14,8	74,0%

*C. reticulata*	C 07	CPEO	25,0	0,4–0,6%
	C 08	CPEO vol. fr. 1	10,8	43,2%
	C 09	CPEO vol. fr. 2	10,1	40,4%
	Total vol. fr.		20,9	83,6%

*C. sinensis*	C 10	CPEO	25,0	0,4–0,6%
	C 11	CPEO vol. fr. 1	12,0	48,0%
	C 12	CPEO vol. fr. 2	10,0	40,0%
	Total vol. fr.		22,0	88,0%

**Table 2:** * Citrus *samples compounds in percentage of total composition.

Compounds	RI	C 01	C 02	C 03	C 04	C 05	C 06	C 07	C 08	C 09	C 10	C 11	C 12	Identification
*α*-Thujene	930				0,6	1,3								a, b, c
*α*-Pinene	939	0,8	0,9		2,3	5,1	2,1	0,8	1,7		0,9	1,3	0,9	a, b, c
Sabinene	975	0,9	1,2						1,2				0,8	a, b, c
*β*-Pinene	976				10,5	19,2	13,7							a, b
Myrcene	991	2,6	2,9	1,8	2,0	3,7	1,9	2,7	4,3	2,2	3,1	3,6	4,1	a, b, c
D-Limonene	1029	72,4	89,6	79,6	37,2	52,7	56,5	80,1	92,8	97,8	83,9	95,0	90,8	a, b, c
*trans*-*β*-ocimene	1050		0,3		0,2	0,3								a, b
*γ*-Terpinene	1060				10,4	13,9	13,4							a, b, c
*α*-Terpinolene	1089		0,3	0,6	0,7	0,9	0,7						1,1	a, b
Nonanal	1101				0,2									a, b
*cis*-limonene oxide	1137												0,4	a, b
Citronellal	1153		0,3	0,5	0,3	0,3								a, b
*α*-Terpineol	1179	0,8	1,0	3,2	0,3		0,5							a, b
Decanal	1202	0,4	0,7	1,9									0,9	a, b
Neral	1238				1,2	0,6	1,5							a, b
Carvone	1243			0,6									0,5	a, b
Lavandulyl acetate	1290				0,9	0,2	1,2							a, b
Citral	1320				2,0	0,9	2,6							a, b
Neryl acetate	1362			0,7	1,2	0,4	1,5							a, b
*α*-Copaene	1377		0,4	1,6										a, b
*β*-Cubebene	1388		0,4	1,7										a, b
*β*-Caryophyllene	1419	0,9	0,9	4,3	0,6		0,8							a, b, c
*α*-Bergamotene	1435				1,0	0,2	1,5							a, b
*α*-Humulene	1456			0,7										a, b
Germacrene D	1485			0,9										a, b, c
Valencene	1496				0,2								0,5	a, b
Bicyclogermacrene	1500				0,1									a, b
*β*-Bisabolene	1506				1,5	0,2	2,2							a, b, c
*δ*-Cadinene	1523			1,4										a, b
Nootkatone	1807	1,1		0,5										a, b
Total		79,8	98,9	100,0	73,4	99,8	100,0	83,5	100,0	99,9	87,9	99,9	99,9	

Sample names according to Table 1; RI = Retention Index; identification method: a = MS, b = RI, and c = comparison with authentic standard.

**Table 3:** Mean percentage (±s.e.) of dead larvae of *Aedes albopictus* in larvicidal bioassays by the 12 *Citrus* samples.

Code	Mean percentage (±s.e.) of dead larvae
C 01	2.0 ± 1.2
C 02	28.0 ± 6.0
C 03	24.0 ± 6.0

C 04	23.0 ± 8.8
C 05	74.0 ± 6.0
C 06	31.0 ± 2.9

C 07	2.0 ± 1.2
C 08	14.0 ± 4.3
C 09	0.0 ± 0.0

C 10	10.0 ± 8.8
C 11	51.0 ± 6.2
C 12	94.0 ± 3.7
DMSO^*∗*^	0.0 ± 0.0

^*∗*^Control.

**Table 4:** Mean number (±s.e.) of landings of *Aedes albopictus* on the uncovered area of the glove per 5 minutes and comparison with the positive control (DEET) and the control (DCM) by using the 12 *Citrus* essential oils (d.f. = 1. *α* = 0.05).

Code	Mean number (±s.e.) of landings/5 min	*P*_DEET_	*P*_DCM_
C 01	32.9 ± 3.9	0.0003^*∗*^	0.0011^*∗*^
C 02	50.6 ± 3.6	0.0003^*∗*^	0.1712
C 03	38.0 ± 1.0	0.0003^*∗*^	0.0008^*∗*^

C 04	23.0 ± 5.3	0.0003^*∗*^	0.0011^*∗*^
C 05	39.0 ± 5.2	0.0003^*∗*^	0.0117^*∗*^
C 06	39.5 ± 7.7	0.0003^*∗*^	0.1275

C 07	36.0 ± 2.7	0.0003^*∗*^	0.0008^*∗*^
C 08	32.6 ± 0.9	0.0003^*∗*^	0.0008^*∗*^
C 09	18.0 ± 3.0	0.0003^*∗*^	0.0008^*∗*^

C 10	49.6 ± 1.9	0.0003^*∗*^	0.0237^*∗*^
C 11	51.0 ± 4.3	0.0003^*∗*^	0.1412
C 12	52.1 ± 5.1	0.0003^*∗*^	0.1267
DEET	0.0 ± 0.0	—	—
DCM	56.0 ± 4.0	—	—

^*∗*^Significant difference.
